# The Association Between Dyslipidemia and Lethality of Suicide Attempts: A Case-Control Study

**DOI:** 10.3389/fpsyt.2019.00070

**Published:** 2019-03-01

**Authors:** Andrea Aguglia, Paola Solano, Gabriele Giacomini, Matilde Caprino, Claudia Conigliaro, Miroslav Romano, Eugenio Aguglia, Gianluca Serafini, Mario Amore

**Affiliations:** ^1^Section of Psychiatry, Department of Neuroscience, Rehabilitation, Ophthalmology, Genetics Maternal and Child Health, University of Genoa, Genoa, Italy; ^2^IRCCS Ospedale Policlinico San Martino, Genoa, Italy; ^3^Department of Experimental and Clinical Medicine, Psychiatric Clinic University Hospital “Gaspare Rodolico, ” University of Catania, Catania, Italy

**Keywords:** suicide, lethality, dyslipidemia, cholesterol, -c reactive protein, metabolic profile, inflammation

## Abstract

Evidence supports the existence of an association between dyslipidemia, psychiatric disorders, and suicide risk due to the effects of altered lipid profiles on serotoninergic neuron membranes. The aim of this study was to investigate the differences in c-reactive protein (CRP), thyroid functioning, total cholesterol, high lipoprotein density cholesterol (HDL-c), low-lipoprotein density cholesterol (LDL-c), and triglycerides (TG) serum levels in low lethality (LLSA) vs. high lethality suicide attempters (HLSA) within 24 h from the suicide attempt and inpatients who never attempted suicide (NAS). After attempting suicide, subjects were admitted to the emergency ward of the IRCCS Ospedale Policlinico San Martino and later to the section of Psychiatry from 1st August 2013 to 31st July 2018. Socio-demographic and clinical characteristics, serum lipids profile, CRP, and thyroid functioning were collected. The sample consisted of 133 individuals with a HLSA, 299 subjects with LLSA, and 200 patients NAS. HLSA subjects were more likely to be males and diagnosed as having a bipolar disorder. Furthermore, HLSA subgroup showed significantly lower total cholesterol and LDL-c levels and higher CRP serum levels compared to LLSA and control group, respectively. LLSA subgroup showed higher HDL-c levels compared to HLSA subgroup (no differences between HLSA and control group were observed). Additionally, the control group reported higher triglycerides levels compared to patients admitted to psychiatric ward for a suicide attempt. Only male gender, having a diagnosis of bipolar disorder, lower total cholesterol, and higher CRP serum levels predicted HLSA. Investigating the relation between dyslipidemia and the severity of suicide attempts may contribute to reveal the complex determinants underlying at-risk behaviors such as suicide, thus playing a relevant role in the possible prevention of this disabling phenomenon.

## Introduction

Suicide and non-fatal suicidal behaviors are major causes of mortality and morbidity worldwide. The World Health Organization (1) estimated that ~800,000 people die from suicide each year and a number from 10 to 20 times higher of individuals attempt suicide, indicating that both suicide and non-fatal suicidal behaviors need to be addressed as a real health priority. The variability in rates of suicidal behaviors within and between countries has been attributed to both population and individual risk factors, including economic status and cultural differences (2) that may significantly affect suicide risk.

Different explanatory models were developed in order to reveal the complex interplay between neurobiological factors such as genetic risk variables, altered serotonergic functioning, and stress responses potentially leading to suicidal behaviors (3, 4). Interestingly, two major dietary lipid classes, cholesterol, and polyunsaturated fatty acids (PUFAs), were significantly associated with higher suicide risk (5, 6). Consistent with the inflammation-related hypothesis of depression and suicidal behavior, C-reactive protein (CRP) blood levels were directly associated with the enhanced risk of attempting and committing suicide (7–9), suggesting that CRP may be a trait marker of suicidal behavior due to its pro-inflammatory effect together with its growing levels during acute inflammation (10). From a genetic perspective, genome-wide association studies (GWAS) identified a region on 2p25 that influences risk for attempting suicide and contains the ACP1 gene (11, 12) and polymorphisms in ACP1 which were found to modulate both protection and predisposition to dyslipidemia (13).

The association between low total cholesterol and cholesterol metabolites serum levels with higher suicide risk has been reported since 1990, when Muldoon et al. initially showed that treatments able to reduce cholesterol levels may attenuate the excess of suicidal behaviors and injury deaths in their sample (14). These results were confirmed by a wide body of literature showing significant associations between altered lipid profiles and higher suicide risk both in patients with specific psychiatric disorders as well as in non-clinical populations (15–21). Recently, Wu et al. conducted a large meta-analysis on 65 epidemiological studies, involving 510.392 participants, and investigated the association between serum lipid levels and “suicidality” subjects defined as individuals presenting suicidal ideation, suicide attempt, having threatened suicide, or death by suicide. Their findings showed that total cholesterol (TC) and low density lipoprotein cholesterol (LDL-c) levels were lower in suicidal patients than non-suicidal patients and healthy controls, high density lipoprotein cholesterol (HDL-c) levels were lower in suicidal patients relative to healthy controls, and triglycerides (TG) levels were lower in suicidal when compared to non-suicidal patients, respectively. Importantly, when the three groups were pooled, lower serum TC was associated with a 112% higher risk of suicidal behaviors (22).

Moreover, subjects who attempted suicide within a month from the blood tests had significantly lower TG and higher HDL-c levels than lifetime suicide attempters and those who never attempted suicide, with TG levels that were negatively associated with current suicidal behavior (23). However, other studies investigating a sample of inpatients with type 1 bipolar disorder and other psychiatric conditions failed to confirm these findings reporting no significant differences in lipid profiles between suicidal and non-suicidal subjects (24–26).

Recent studies investigated the role of cholesterol levels in violent vs. non-violent suicide attempts and showed that the former was significantly associated with lower cholesterol serum levels of ~30% than the latter (27–29). Moreover, two post-mortem studies showed significantly lower cholesterol levels in the pre-frontal-cortex (PFC) of violent suicide attempters and significantly higher cholesteryl-ester-hydrolase (LIPA) expression in violent suicide attempters when compared with non-violent suicide attempters (30, 31).

Given this background, in this study we investigated the differences in CRP, thyroid functioning, TC, HDL-c, LDL-c, and TG serum levels between low-lethality (LLSA) vs. high-lethality suicide attempts (HLSA) within 24 h from the suicide attempt and inpatients who never attempted suicide (NAS).

According to this main objective, we tested the following hypothesis: (a) lower total cholesterol, HDL-c, LDL-c, and TG serum levels determine HLSA instead of LLSA and NAS; (b) CRP levels are higher in HLSA instead of LLSA and NAS.

## Materials and Methods

### Sample

The present study was conducted in a sample of patients who were recruited at the section of Psychiatry of the IRCCS Ospedale Policlinico San Martino—Department of Neuroscience, Rehabilitation, Ophthalmology, Genetics, Maternal, and Child Health, University of Genoa, Italy, from 1st August 2013 to 31st July 2018.

The inclusion criteria were: (a) hospitalization in an emergency psychiatric ward for a suicide attempt; (b) aged over 18 years old; (c) the willingness to participate in the study by signing a written informed consent. The exclusion criteria were: (a) pregnancy or having just given birth; (b) having a positive history of acute neurological injury, such as neurodegenerative illnesses, mental retardation, loss of consciousness related to the presence of severe neurological conditions; (c) the assumption of lipid-lowering agents; (d) the refusal or inability to provide a valid consent prior to participate in the study.

A control group was also included in the sample and it was represented by admitted patients without a history of current and/or lifetime suicide attempts. The control group was matched for age, gender, occupational/marital status, and diagnosis to avoid any bias. We initially screened a sample of 703 patients; however, only 632 subjects voluntarily accepted to participate in the study by signing a written informed consent, the remaining individuals were lost due to lack of serum data or because they did not sign the required informed consent.

The study design was reviewed and approved by the local ethic committee.

### Assessments and Procedures

Socio-demographic and clinical characteristics of the recruited patients were investigated during hospitalization through the standardized clinical chart and lifetime computerized medical record, used in Psychiatric Unit. The following patients' domains: age, gender, marital and occupational status, education level, suicide attempts, and suicide method were carefully investigated.

All available information have been cross-referred.

Psychiatric diagnoses were evaluated and set according to Diagnostic and Statistical Manual of Mental Disorders, fifth edition (DSM 5) (32). Clinical evaluations were carried out by expert clinicians and carefully reviewed by a senior psychiatrist (with at least 10 years of clinical experience in inpatient clinical setting). If patients had more than one psychiatric diagnosis, the principal psychiatric condition as diagnosed by the treating psychiatrist, was recorded. According to previous published studies (33, 34), we grouped the diagnosis in four main categories: bipolar and related disorders, depressive disorders, schizophrenia, and related disorders, other psychiatric disorders.

Based on Schrijvers et al., we considered suicide as a process, for which suicidal behaviors can be broken down chronologically into “component parts,” beginning with the development of suicidal ideation, that progresses to planning, then putting thoughts and plans into action via attempts, and, if successful, culminating in completed suicide (35).

The term “suicidal lethality” has not yet been defined outside health literature. Beyond one publication describing suicide lethality as the lethality of the chosen suicide method (36), some theorists like Shneidman and Joiner conceptually identified suicide lethality “as a key ingredient of serious suicidality” (37, 38). We adopted Joiner's definition of suicide lethality, defined as “the acquired ability to enact lethal self-injury” (38). Within suicide lethality, the only individual intent is to perish as a result of the lethality of self-inflicted actions. Methods of suicide attempt were dichotomized in terms of lethality. Therefore, a high-lethality suicide attempt was defined as a suicide attempt that warranted hospitalization for at least 24 h and either treatment in a specialized unit (including intensive care unit, hyperbaric unit, or burn unit), surgery under general anesthesia, or extensive medical treatment (beyond gastric lavage, activated charcoal, or routine neurological observations), including antidotes for drug overdoses, telemetry, or repeated tests or investigations. Conversely, a low-lethality suicide attempt was defined as a suicide attempt that did not meet these criteria (39–46).

A routine blood examination was usually performed at hospital admission for all patients as a part of the clinical management routine. Blood samples were taken between 7:00 and 8:30 a.m. after patients had fasted for at least 10 h and after a psychiatric evaluation; patients who were not fasting were rescheduled. Blood exams included TC, TG, HDL-c, LDL-c, CRP serum levels, and TSH Reflex. Blood samples were drawn during the hospitalization in the Psychiatric Clinic and examined in the laboratory analysis of IRCCS Ospedale Policlinico San Martino, Genoa, Italy.

### Statistical Analysis

All statistical analyses were performed using SPSS version 22.0 (IBM Corp., Armonk, NY, USA) with the value of statistical significance which was set at *p* < 0.05.

Socio-demographic and clinical characteristics of the subjects were represented as mean and standard deviation (SD) for continuous variables and as frequency and percentage regarding categorical variables. The Kolmogorov-Smirnov test was conducted to confirm whether all the investigated sample variables followed the normal distribution.

Firstly, the sample was divided in two subgroups according to the presence/absence of current suicide attempt. A statistical comparison between patients with and without current suicide attempt was performed to examine whether there were differences in terms of socio-demographic and diagnostic features. Thus, in order to avoid statistical bias, the two subgroups were matched for age, gender, marital/occupational status, and psychiatric diagnoses.

Subsequently, the subgroup of patients admitted for a current suicide attempt was divided according to the lethality of suicide attempts, identifying a subgroup with high-lethality of suicide attempts and a subgroup with low-lethality of suicide attempts. In order to analyze differences between these three subgroups, we used the Pearson χ2 test with Yates correction for the comparison of categorical variables, and ANOVA for continuous variables.

Lastly, a multinomial regression model was performed to detect the variables associated with the lethality of suicide attempt (dependent variable) and each of the other independent variables previously found associated in the statistical analyses. The probability of entering the equation was set at 0.05.

## Results

In our study we recruited a total sample of 632 patients, with a mean age of 49.69 ± 18.97 years old. Of the total sample, 478 subjects were females (75.6%) with an educational level of 11.06 ± 3.28 years. Four hundred and thirty-two subjects were recruited in the case-group and 200 in the control group. There were no statistically significant differences in socio-demographic and clinical characteristics (i.e., gender, age, marital status, educational level, working status, psychiatric diagnoses and pharmacological treatment) between the subgroup of patients who attempted suicide and the subgroup of patients who never attempted suicide (control group). Socio-demographic and clinical characteristics of the included subjects are summarized in Tables 1, 2.

**Table 1:** Socio-demographic characteristics in the total sample and in the two subgroups.

	**Total sample (*N* = 632)**	**Suicide attempt (*N* = 432)**	**No suicide attempt (*N* = 200)**	***t/χ2***	***df***	***p***
Gender (female), *N (%)*	478 (75.6)	333 (77.1)	145 (72.5)	1.558	1	0.212
Age (years), *mean ± SD*	49.69 ± 18.97	49.13 ± 20.16	50.89 ± 16.11	1.086	630	0.278
Education level, *mean ± SD*	11.06 ± 3.28	11.15 ± 3.27	10.86 ± 3.30	−1.046	630	0.296
**MARITAL STATUS**, ***N (%)***						
Single	296 (46.8)	195 (45.1)	101 (50.5)	4.672	3	0.197
Married	131 (20.7)	93 (21.5)	38 (19.0)			
Divorced	147 (23.3)	98 (22.7)	49 (24.5)			
Widowed	58 (9.2)	46 (10.6)	12 (6.0)			
Working status, *N (%)*	177 (28.0)	121 (28.0)	56 (28.0)	0.000	1	0.998

**Table 2:** Clinical characteristics in the total sample and in the two subgroups.

	**Total sample (*N* = 632)**	**Suicide attempt (*N* = 432)**	**No suicide attempt (*N* = 200)**	***t/χ2***	***df***	***p***
**DIAGNOSIS**, ***N (%)***						
Bipolar and related disorders	206 (32.6)	142 (32.9)	64 (32.0)	4.434	3	0.218
Schizophrenia and related disorders	63 (10.0)	37 (8.6)	26 (13.0)			
Depressive disorders	202 (32.0)	146 (33.8)	56 (28.0)			
Others	161 (25.4)	107 (24.7)	54 (27.0)			
**PHARMACOLOGICAL TREATMENT**, ***N (%)***						
Antipsychotics	252 (58.3)	175 (40.5)	77 (38.5)	0.230	1	0.631
Mood stabilizers	303 (70.1)	202 (46.8)	101 (50.5)	0.766	1	0.381
Antidepressants	165 (38.2)	110 (25.5)	55 (27.5)	0.294	1	0.588
Others	108 (25.0)	82 (19.0)	26 (13.0)	3.452	1	0.063
Drug-free	55 (12.7)	40 (9.3)	15 (7.5)	0.533	1	0.466
Total Cholesterol, *mean ± SD*	178.97 ± 42.50	174.14 ± 45.17	189.41 ± 33.86	4.257	630	<0.001
LDL Cholesterol, *mean ± SD*	117.23 ± 36.47	113.11 ± 37.88	126.15 ± 31.52	4.235	630	<0.001
HDL Cholesterol, *mean ± SD*	51.55 ± 17.11	52.08 ± 17.39	50.43 ± 16.50	−1.128	630	0.260
Tryglicerides, *mean ± SD*	122.63 ± 69.07	114.32 ± 57.38	140.58 ± 86.73	4.514	630	<0.001
TSH reflex, *mean ± SD*	2.33 ± 1.92	2.30 ± 1.89	2.40 ± 1.99	0.629	630	0.529
CRP, *mean ± SD*	10.63 ± 23.63	13.52 ± 27.86	4.37 ± 5.61	−4.599	630	<0.001

Regarding patients admitted for a suicide attempt, 133 individuals (30.8%) committed a HLSA while 299 subjects (69.2%) carried out a LLSA, respectively. The prevalence of the method used to attempt suicide is shown in Table 3.

**Table 3:** Type of suicide according to lethality.

	**Total sample (*N* = 432)**
Suicide Attempt, *N (%)*	432 (68.4)
High lethality	133 (30.8)
Low lethality	299 (69.2)
**TYPE OF SUICIDE ATTEMPT (*****N*** **=** **432)**, ***N (%)***	
Drug intoxication	284 (65.7)
Defenestration	40 (9.3)
Drowning	1 (0.2)
Weapon	2 (0.3)
Stabbing	9 (2.1)
Burn/Gas/Caustic	26 (6.0)
Strangling	11 (2.5)
Cuts	59 (13.7)

Considering socio-demographic and clinical features within the three subgroups, the HLSA subgroup was significantly associated with male gender (38.3 vs. 16.1 vs. 27.5%, *p* < 0.001) and diagnosis of bipolar disorder (41.4 vs. 29.1 vs. 32.0%, *p* = 0.007), compared to LLSA, and control group, respectively.

When the three subgroups were compared, the HLSA subgroup showed significantly lower total cholesterol levels (151.08 ± 40.90 vs. 184.40 ± 43.21 vs. 189.41 ± 33.88, *p* < 0.001) and LDL-c levels (99.55 ± 33.25 vs. 119.15 ± 38.30 vs. 126.15 ± 31.52, *p* < 0.001), and higher CRP serum levels (24.18 ± 38.69 vs. 8.78 ± 19.66 vs. 4.37 ± 5.61, *p* < 0.001) compared to LLSA and control group, respectively.

Furthermore, the LLSA subgroup showed higher HDL-c levels compared to HLSA subgroup (54.64 ± 16.59 vs. 46.31 ± 17.82, *p* < 0.001) (no differences between HLSA and control group were observed). Additionally, the control group reported higher triglycerides level compared to patients admitted for a suicide attempt. No differences in triglycerides levels between HLSA and LLSA were found). Additional differences are shown in Table 4.

**Table 4:** Comparison among three subgroups according to the lethality of suicide attempt.

	**High lethality (*N* = 133)**	**Low lethality (*N* = 299)**	**Controls (*N* = 200)**	***F***	***p***	***post-hoc* (Bonferroni)**
Gender (male), *N (%)*	51 (38.3)	48 (16.1)	55 (27.5)	26.380	<0.001	
Age (years), *mean ± SD*	49.62 ± 20.69	48.91 ± 19.95	50.89 ± 16.11	0.653	0.521	H = L = C
Education level, *mean ± SD*	10.93 ± 3.15	11.24 ± 3.32	10.86 ± 3.30	0.963	0.382	H = L = C
**MARITAL STATUS**, ***N (%)***						
Single	62 (46.6)	133 (44.5)	101 (50.5)	5.477	0.484	
Married	30 (22.6)	63 (21.0)	38 (19.0)			
Divorced	29 (21.8)	69 (23.1)	49 (24.5)			
Widowed	12 (9.0)	34 (11.4)	12 (6.0)			
Working status, *N (%)*	36 (27.1)	85 (28.4)	56 (28.0)	0.085	0.959	
**DIAGNOSIS**, ***N (%)***						
Bipolar and related disorders	55 (41.4)	87 (29.1)	64 (32.0)	17.603	0.007	
Schizophrenia and related disorders	16 (12.0)	21 (7.0)	26 (13.0)			
Depressive disorders	41 (30.8)	105 (35.1)	56 (28.0)			
Others	21 (15.8)	86 (28.8)	54 (27.0)			
Total Cholesterol, *mean ± SD*	151.08 ± 40.90	184.40 ± 43.21	189.41 ± 33.88	41.921	<0.001	L = C>H
LDL Cholesterol, *mean ± SD*	99.55 ± 33.25	119.15 ± 38.30	126.15 ± 31.52	23.600	<0.001	L = C>H
HDL Cholesterol, *mean ± SD*	46.31 ± 17.82	54.64 ± 16.59	50.43 ± 16.50	11.946	<0.001	L>H = C
Triglycerides, *mean ± SD*	122.13 ± 59.78	110.84 ± 56.03	140.58 ± 86.73	11.480	<0.001	C>H = L
TSH reflex, *mean ± SD*	2.04 ± 1.89	2.42 ± 1.88	2.40 ± 1.99	1.988	0.138	H = L = C
CRP, *mean ± SD*	24.18 ± 38.69	8.78 ± 19.66	4.37 ± 5.61	32.808	<0.001	H>L = C

When the multinomial regression was performed, male gender, diagnosis of bipolar disorder, lower total cholesterol level, and higher CRP serum levels predicted HLSA (Table 5).

**Table 5:** Relationship between potential explanatory variables and lethality of suicide attempts: results from the logistic regression analysis.

**Step**	**Variables**	**T**	**E.S**.	**Wald**	**df**	***p***	**Exp(B)**	**95% CI for EXP**
Step 1	Gender	−0.684	0.274	6.250	1	0.012	0.504	0.295–0.863
Step 2	Diagnosis	0.498	0.252	3.903	1	0.048	1.645	1.004–2.695
Step 3	Total cholesterol	−0.022	0.006	15.377	1	<0.001	0.979	0.968–0.989
Step 4	LDL cholesterol	0.004	0.006	0.479	1	0.489	1.004	0.992–1.017
Step 5	HDL cholesterol	−0.014	0.008	3.483	1	0.062	0.986	0.971–1.001
Step 6	Triglycerides	0.004	0.002	3.099	1	0.078	1.004	1.000–1.008
Step 7	c-reactive protein—CRP	0.017	0.005	13.844	1	<0.001	1.017	1.008–1.026
	Constant	3.388	0.759	19.950	1	<0.001		

## Discussion

The present study tested the association between lipid profile, CRP levels, and thyroid functioning in a relatively large sample of psychiatric inpatients who committed HLSA (133 subjects) vs. psychiatric inpatients who carried out LLSA (299 subjects) and a control group of psychiatric inpatients who never attempted suicide (200 subjects).

HLSA were more likely to be males and affected by bipolar disorder. This is consistent with previous findings showing that subjects with bipolar disorder presented a higher risk for attempting and committing suicide (47–49) and supported the higher lethality suicide among males irrespective from their psychiatric diagnosis (46, 49–51).

Our study showed that HLSA was clearly associated with lower total cholesterol and LDL-c, and higher CRP levels when compared with LLSA and controls. To the best of our knowledge, no previous studies investigated the association between suicide lethality and lipid profiles, though our results may be explained in the light of previous evidence showing that violent methods to attempt suicide were associated with lower total cholesterol levels (27).

The well-known cholesterol-serotonin hypothesis (52) may help to explain these results as lower total cholesterol levels may foster higher central neuroinflammation, thus altering the serotonergic system and leading to higher aggressiveness and impulsivity, especially among males (53). In the central nervous system, serotonin plays a role in the suppression of aggressive and harmful behaviors. There are several theories that may explain the potential effect of serum lipid profile (in particular cholesterol levels) on violent conduct and suicide risk. The most blamed mechanism is the reduction of brain serotonergic activity which is associated with the risk of attempting suicide. It has been hypothesized that cholesterol levels are associated with the lipid micro viscosity of serotonin receptors and transporters. Since reliable evidence shows that circulating levels of cholesterol—those that may be detected by routine blood tests as those used on the patients in the study—correlate with the role of stabilizer of cellular membrane functioning (6), and membrane cholesterol exchanges freely with cholesterol in the surrounding medium, low membrane cholesterol decreases the number of serotonergic receptors through the decreased lipid micro viscosity of the serotonergic receptor on the neuronal membrane (53, 54). This process could lead to a poorer suppression of impulsive and violent behaviors, such as suicidal behaviors (54). As a matter of fact, cholesterol is crucial for membrane stability and neurotransmission that include the alteration of membrane lipid raft structure by the proportions of cholesterol and n-3 PUFAs, affecting the functioning of membrane-bound proteins including serotonin receptors and transporters, and toll-like receptors (6, 55). Therefore, low levels of cholesterol might be responsible of increased n-6:n-3 PUFA ratio, thereby promoting neuroinflammation as n-3 PUFAs tend to exert anti-inflammatory properties, while n-6 PUFAs levels tend to show a pro-inflammatory activity, and disinhibit, albeit indirectly, two inflammatory intermediates such as nuclear factor kappa-light-chain-enhancer of activated B cells (NFκB) and peroxisome proliferator activated receptors (PPARs), respectively (56). The abnormal monoaminergic neurotransmission as well as neuroinflammation are two leading mechanisms which are evoked as biological pathways underlying suicidal behavior (6, 9, 55). Lower cholesterol levels are associated with greater impulsivity of suicide attempts and violent methods due to their effects on the serotonergic system. Indirect evidence suggests an association between attempt lethality and low-cholesterol levels on the basis of the relation between lethality and the choice of violent methods to attempt suicide (29).

Although, to the best of our knowledge, no studies investigated directly the association between CRP, cholesterol, and cholesterol metabolites serum levels and lethality of suicide attempts, interestingly, a recent study hypothesized a bridge between the well-known cholesterol metabolism process with its associated molecular pathways and the neurobiological underpinnings of suicide risk, by showing that the relation between total unesterified cholesterol and suicide risk was significantly mediated by ABCA-1-specific cholesterol efflux capacity (57).

Conversely, our results show no differences in total cholesterol and LDL-c levels between LLSA and controls, although ANOVA did not confirm these findings. The lack of differences concerning lipid profiles of LLSA and controls could be explained in the light of the method lethality used to attempt suicide. For instance, Lalovic et al. (30) reported no significant differences in cholesterol content between suicide victims and controls in specific brain regions such as the frontal cortex, amygdala, and hippocampus. However, when suicides were classified as violent or non-violent according to the used method, violent suicides were found to have lower gray-matter cholesterol content in the frontal cortex compared to non-violent suicides. Other authors (58), albeit in a small sample, reported no difference in the levels of total cholesterol and triglycerides among attempters and non-attempters.

Our findings show that TG were significantly higher in the control group than in LLSA and HLSA subgroups, among which no significant differences were reported. This is consistent with previous studies showing lower TG levels among suicide attempters when compared with controls without a positive history of suicide attempts (17, 20–22). Lower TG levels were reported in subjects who attempted suicide in the month before the survey compared with subjects who had suicidal ideation in the month before the survey and never suicidal controls (23).

Moreover, our findings do not show any differences regarding TG levels in LLSA and HLSA subgroups; to date, no previous studies investigated the possible association between the lethality of suicide attempts and TG levels. There are studies in the current literature that reported no differences in TG levels among violent vs. non-violent suicide attempts, though they did not consider the lethality of suicide attempts (25, 59).

Our study should be considered in the light of the following limitations; firstly, this is a cross-sectional study, and we cannot assess whether a decrease in TC or TG may have caused a mood episode with active suicidal ideation leading to suicide attempts, or if the presence of a mood episode originated a loss of appetite and a consequent loss of weight altering lipid profiles. Thus, given the main nature of this study, we could not evaluate the direct causal relation between suicidal behaviors and lipid profile. Moreover, our results could not be adjusted for the psychopharmacological medications that both cases and controls were taking when assessed and this may have influenced our findings. However, subjects taking lipid-lowering agents were not included in the sample. Thirdly, a detailed medical history, including careful information about the body mass index (BMI) was not available. Neither blood pressure nor glycaemic values were collected and, consequently, included in the analysis. However, we only included those subjects with stable clinical conditions apart from what was related to suicide attempts.

## Conclusions

Our data suggest that low total cholesterol serum levels may increase the risk of HLSA and low triglycerides serum levels increase suicide risk—as well as low TC levels do—but they do not influence the lethality of the attempt. To the best of our knowledge, no previous studies have investigated TC and TG levels in respect to the lethality of suicide attempt. Therefore, further studies should focus on this association in order to confirm these preliminary results and shed light on the complex neurobiological mechanisms underlying suicidal behaviors.

## Author Contributions

AA: supervision data collection, writing protocol, statistical analyses, writing original draft; PS: writing original draft, designed the study; GG: writing protocol, conceived, and designed the study; MC, CC, and MR: data collection, revision of data literature; EA and MA: review and editing of the original draft, scientific advisor of the project; GS: designed the study, review, and editing of the original draft. All authors approved of the final draft of the manuscript before submission.

### Conflict of Interest Statement

The authors declare that the research was conducted in the absence of any commercial or financial relationships that could be construed as a potential conflict of interest.
